# Supplementary material for: A new genus and species of frog from the Kem Kem (Morocco), the second neobatrachian from Cretaceous Africa
Source: PeerJ. 2022 Jul 15;10:e13699. doi: 10.7717/peerj.13699 (PMC9291016; doi:10.7717/peerj.13699)
Supplement: Data S1 [file peerj-10-13699-s001.docx]

**A new genus and species of frog from the Kem Kem (Morocco), the second neobatrachian from Cretaceous Africa**

Supplemental Data 1

Alfred Lemierre^1^, David Blackburn^2^

^1.^ CR2P- Centre de recherche en Paléontologie-CNRS/MNHN/Sorbonnes Université, Bâtiment de Géologie, 43 rue Buffon, Paris, 75005, France, [alfred.lemierre@edu.mnhn.fr](mailto:alfred.lemierre@edu.mnhn.fr),

^2.^ Department of Natural History, Florida Museum of Natural History, University of Florida, Gainesville, FL 32611, USA, dblackburn@flmnh.ufl.edu.

Table S1. List of CT-scan and 3D models of UCRC-PV94 ̶ 98, 101, 103 ̶ 105

Appendix S1 Characters list for analyses. Symbol: *, hyperossified character.

**Skull**

1. Skull roof, orbital region, minimum width relative to maximum antorbital width: (0) less than 1/4, (1) between 1/4 and 1/3, (2) more than 1/3.

2. Skull roof, orbital region, shape: (0) posterior and anterior ends equally wide, (1) anteriorly wider than posteriorly, (2) posteriorly wider than anteriorly.

*3. Nasals, relation to one another: (0) well separated, (1) slightly separated or in minimal contact, (2) in contact throughout most of their medial margins, or completely fused.

4. Nasal, position of anterior end relative to anterior tip of maxilla: (0) posterior, (1) same level, (2) anterior.

*5. Frontoparietals, relation to one another: (0) separate along entire length, (1) partially sutured or in contact only partially, may be partially fused, (2) sutured along entire length, (3) azygous.

*6. Frontoparietal, supraorbital flange: (0) absent, (1) present.

*7. Occipital artery pathway, relation to skull roof: (0) superficial, (1) in open groove, (2) enclosed in canal.

*8. Squamosal-frontoparietal relationship: (0) separated, (1) contact between lamella alaris of squamosal and frontoparietal, with bony bridge arching over the otic capsule to form a post temporal fenestra (*sensu* Lynch 1971: 47), (2) contact between lamella alaris and frontoparietal on the dorsal surface of the otic capsule, no post temporal fenestra present, (3) contact restricted to the otic plate of otic ramus and frontoparietal (e.g. *Bufo*).

*9. Squamosal, otic ramus, otic plate (= ramus paroticus of Roček 2003): (0) reduced or absent, (1) small otic plate overlapping only most lateral portion of crista parotica, (2) otic plate extensively overlapping prootic.

*10. Squamosal, zygomatic ramus: (0) reduced, (1) moderately developed, free distal end, (2) well developed, articulated with maxilla.

11. Squamosal-nasal relationship: (0) not in contact, (1) in contact.

12. Pterygoid, anterior ramus, anterior extent: (0) reaching planum antorbitale, (1) not reaching planum antorbitale*.*

13. Pterygoid, anterior ramus, position relative to maxilla: (0) medial, (1) dorsal.

14. Pterygoid, medial ramus, configuration: (0) short, without bony contact with otic capsule, (1) moderately developed, contact with otic capsule, (2) well developed, broad contact, overlapping or sutured with parasphenoid ala.

15. Pterygoid, anterior ramus, ventral flange: (0) absent, (1) present.

16. Pterygoid, anterior ramus, dorsal flange: (0) absent, (1) present.

17. Parasphenoid, cultriform process, anterior extent relative to antorbital plane: (0) well posterior, (1) nearly same level, (2) anterior.

18. Parasphenoid, cultriform process, longitudinal ridge on ventral surface: (0) absent, (1) present.

19. Parasphenoid, alae, orientation in ventral aspect: (0) angled anteriorly, (1) perpendicular to cultriform process, (2) angled posteriorly.

20. Parasphenoid, alae, keels on ventral surface: (0) absent, (1) present.

21. Vomer: (0) absent, (1) present.

22. Vomer, teeth: (0) present, (1) absent.

23. Vomer, teeth, position relative to planum antorbitale: (0) clearly anterior, (1) same level.

24. Vomer, teeth, disposition: (0) straight rows, perpendicular to skull midline, (1) straight rows, divergent posteriorly, (2) straight rows, divergent anteriorly, (3) rows arched anteriorly, (4) rows arched posteriorly, (5) few teeth on a round bulging patch.

25. Vomer, anterior process, extent relative to maxillary arch: (0) reaching maxillary arch, (1) not reaching maxillary arch.

26. Vomer, postchoanal process: (0) not distinct, (1) distinct but short, (2) well developed, long, bordering the entire posterior margin of choana.

27. Palatine, as a discrete element: (0) absent, (1) present.

28. Palatine, odontoids or ridges on ventral surface: (0) absent, (1) present.

29. Sphenethmoid, dorsal exposure between nasals and frontoparietals: (0) absent, (1) present.

30. Sphenethmoid, ventral configuration: (0) single, (1) divided.

31. Sphenethmoid, dorsal configuration: (0) single, (1) divided.

32. Sphenethmoid, orbitonasal foramen: (0) completely bound in bone, (1) incompletely bound in bone or bound in cartilage.

33. Sphenethmoid, planum antorbitale, mineralization: (0) mostly cartilaginous, (1) well ossified, at least one half of planum.

34. Nasal capsule, septum nasi, mineralization: (0) mostly cartilaginous, (1) partially ossified, (2) completely ossified.

35. Nasal capsule, septum nasi, width: (0) wide, nasal capsules medially separate, (1) narrow, nasal capsules medially close.

36. Cartilaginous roofing of cavum cranii, configuration: (0) completely open, (1) taenia tecti transversalis present only, (2) taeniae tecti transversalis and medialis present, (3) tectum parietale present, (4) almost completely chondrified, (5) taeniae tecti medialis present only.

37. Neurocranium, lateral wall at the level of the optic foramen, mineralization: (0) cartilaginous (1) partially or completely ossified.

38. Crista parotica, degree of ossification: (0) mostly cartilaginous, (1) ossified at least one half.

39. Crista parotica, distal expansion: (0) absent, (1) present.

40. Exoccipital, occipital condyles, shape: (0) not stalked, (1) stalked.

41. Tympanic annulus: (0) absent, (1) present.

42. Columella: (0) absent, (1) present.

43. Teeth on premaxilla and maxilla: (0) absent, (1) present.

44. Tooth crown, number of cuspids: (0) two, tooth bicuspid, labial cusp may be present as low blade, (1) one, tooth monocuspid.

45. Premaxilla, pars palatina, palatine process, configuration: (0) distinct, projecting lingually, (1) investing alary process proximally, distinct pointed distal (posterior) tip, (2) entire process investing alary process, blunt distal tip distinct or absent.

46. Premaxilla, pars palatina, lateral end, configuration: (0) not expanded lingually nor projected posteriorly (1) expanded lingually, but less than palatine process, and slightly projected posteriorly, (2) broad but indistinct from the rest of the pars palatina, not projected posteriorly, (3) expanded lingually as much as palatine process, not projected posteriorly, (4) distinctly projected towards maxilla forming an extensive suture, slightly or not expanded lingually, a discrete lingual process may be present.

47. Premaxilla, alary process, orientation: (0) anterodorsal, (1) dorsal, (2) posterodorsal.

48. Premaxilla, contact with maxilla in labial aspect: (0) maxilla abuts premaxilla, (1) maxilla overlaps premaxilla, short anteroventral process, (2) maxilla overlaps premaxilla, long anterodorsal process, (3) maxilla overlaps premaxilla, long anteroventral process, (4) maxilla overlaps entire premaxilla.

*49. Maxilla, pars facialis in the orbital region, shape: (0) decreases gradually in height in the orbital region, (1) decreases abruptly in height in the orbital region, (2) high along the orbital region but decreases posteriorly, (3) high throughout most of its length.

*50. Maxilla, pars facialis external surface, aspect: (0) smooth, (1) slightly sculptured, only some ridges present, (2) heavily ornamented, usually tuberculated or pitted, sculpture lies over the external surface of the pars dentalis, (3) heavily ornamented, usually tuberculated or pitted, sculpture lies mainly in a deeper plane than that of the protruding external surface of the pars dentalis.

51. Maxilla, pars facialis, anterodorsal process: (0) absent or reduced (i.e., reaching more posteriorly than the anteroventral margin of the bone) (e.g., Litoria australis), (1) reaching more anteriorly than the anteroventral margin of the bone but not well differenciated from the rest of the pars facialis (e.g., Calyptocephalella), (2) present, long, projecting anterodorsally and well differenciated from the rest of the pars facialis (e.g., Ceratophrys).

52. Maxilla, pars palatina, orientation at the level of the planum anteorbitale: (0) forming a straight angle with the pars dentalis, (1) forming a wide angle with the pars dentalis, (2) forming a 180º angle with the pars dentalis and parallel to the internal surface of pars facialis.

53. Quadratojugal, pars jugalis: (0) absent, (1) present.

*54. Quadratojugal, dorsal process: (0) absent, (1) present.

*55. Quadratojugal, lateral process: (0) absent, (1) present.

56. Mentomeckelian bone: (0) absent, (1) present.

57. Mentomeckelian bone, lateral process: (0) absent, (1) present.

58. Dentary, fang-like laminar projections: (0) absent, (1) present.

59. Fang-like, symphyseal ectopic ossifications: (0) absent, (1) present.

60. Angulosplenial, coronoid process, shape: (0) poorly developed, low, (1) well developed, distinct process, (2) very well developed, long lamina.

61. Lower jaw, articulation with skull, position: (0) anterior to occiput, but lateral to otic capsule, (1) same level of occiput, (2) well posterior to occiput, (3) well anterior, anterior to otic capsule.

**Hyobranchial skeleton**

62. Hyoid, hyalia, general configuration: (0) complete, (1) incomplete distally, (2) incomplete proximally.

63. Hyoid, anterior process of hyale: (0) absent, (1) present.

64. Hyoid, process lateral to anterior process of hyale: (0) absent, (1) present.

65. Hyoid plate, anterolateral (alary) processes: (0) absent, (1) present.

66. Hyoid plate, anterolateral (alary) processes), shape: (0) acuminate, (1) narrow stalk and dilated distally, (2) wing-like, (3) broad stalk and dilated distally, (4) confluent with hyoid plate.

67. Hyoid plate, posterolateral process: (0) absent, (1) present.

68. Hyoid plate, posteromedial process, ossification: (0) cartilaginous stalk abuts hyoid plate, (1) ossification reaches hyoid plate, (2) ossification invades hyoid plate.

69. Hyoid plate, posteromedial process, flange on medial side: (0) absent, (1) present.

70. Hyoid, posterior projection on hyoid plate: (0) absent, (1) present.

71. Hyoid plate, shape: (0) wide, width greater than, or equal to, length, (1) narrow, longer than wide.

72. Hyoid plate, calcification: (0) absent, (1) present.

73. Parahyoid bone: (0) absent, (1) present.

**Vertebra Column**

74. Presacral vertebrae, number (irrespective of fusions): (0) more than eight, (1) eight or less.

75. Trunk vertebrae, centrum formation: (0) perichordal, (1) epichordal.

76. Atlas, cotyle arrangement: (0) fully confluent (Type III), (1) juxtaposed (Type II), (2) widely separated (Type I).

77. Atlas, cotyle position relative to neural canal: (0) completely ventral, (1) ventrolateral.

78. Vertebrae I and II relationship: (0) completely separate, (1) neural spine I flattened, overlapping and fused to neural arch II, (2) completely fused.

79 Posteriormost presacral vertebra, centrum configuration: (0) spool-like, (1) opisthocoelous, (2) procoelous, (3) biconcave.

80. Posterior four presacrals, neural arches, imbrication: (0) present, (1) absent.

81. Anterior presacrals, neural spines: (0) absent or low, (1) high, dorsally directed, (2) high, posteriorly directed.

82. Vertebra VI, transverse processes, posterior margin orientation: (0) nearly perpendicular to longitudinal axis or slightly posterior, (1) moderately forward, (2) markedly forward.

83. Vertebra IV, transverse processes, mediolateral length relative to that of sacral diapophyses: (0) nearly equal or slightly longer, (1) much longer (25% or more).

84. Posteriormost presacral vertebra, transverse processes, mediolateral length relative to that of sacral diapophyses: (0) clearly shorter, (1) equal or subequal.

85. Vertebra III, transverse processes/associated ribs, uncinate processes: (0) present, (1) absent.

86. Sacral diapophyses, distal expansion in dorsal view, evaluated as the distal length (DL) relative to the maximum distance between distal margins of diapophyses (MW) = sacral width of Trueb & Tyler (1974): (0) widely expanded (DL ≥ ½ MW) (1) moderately expanded (½ MW > DL ≥ ¼ MW), (2) slightly expanded or narrow (DL < ¼ MW).

87. Sacral diapophyses, distal ends in lateral view: (0) distinctly flattened (height < ½ anteroposterior length, (1) roughly rounded (height nearly ½ length).

88. Sacral diapophyses, anterior margin, orientation with respect to longitudinal axis in dorsal view: (0) posterolateral, (1) nearly perpendicular, (2) anterolateral.

89. Sacral diapophyses, general orientation in posterior aspect, with respect to the horizontal plane, (0) nearly horizontal or slightly angled dorsally, (1) strongly angled dorsally, forming an angle of 15° or larger.

90. Sacral vertebra, articulation with urostyle: (0) synchondrotic, notochord persistent, (1) bicondylar, (2) monocondylar, (3) fused.

91. Urostyle, transverse processes: (0) present, (1) absent.

92. Urostyle, length relative to presacral portion of vertebral column: (0) nearly as long, or longer, (1) clearly shorter (85% or less).

93. Urostyle, dorsal crest: (0) absent or low ridge, (1) moderate or high, but extending only throughout the anterior one half of urostyle, (2) well developed, high and extending along most of the urostyle.

94. Urostyle, anterodorsal process at anterior end of dorsal crest: (0) absent or poorly developed, (1) distinct, well developed.

95. Urostyle, shape in lateral view: (0) straight, (1) arched.

96. Free ribs in adults: (0) absent, (1) present.

97. Free intervertebral discs in subadults: (0) absent, (1) present.

98. Dorsal shield formed by medial or medial and lateral elements joined to each other and to the vertebrae by ligaments: (0) absent, (1) present.

99. Dorsal shield formed by at least medial elements continuous with the neural spines: (0) absent, (1) present.

**Pectoral girdle**

100. Pectoral girdle, omosternum: (0) absent, (1) present.

101. Pectoral girdle, omosternum, degree of ossification: (0) completely cartilaginous, (1) with ossified style.

102. Pectoral girdle, sternum, degree of ossification: (0) completely cartilaginous or irregularly calcified, (1) with an ossified style.

103. Pectoral girdle, sternum, shape: (0) short and wide, semicircular to rhomboid, (1) proximally bifurcated in long projections, (2) long and distally rounded, (3) inverted mushroom, style short and wide, (4) anchor or arrow, style long and narrow, (5) broad, longer than wide, with posterior notch, (6) narrow style, distally bifurcated, (7) wider posteriorly than anteriorly, irregular posterior margin.

104. Pectoral girdle, epicoracoids in the coracoideal region, relation to one another: (0) overlapping, (1) not overlapping.

105. Coracoid, sternal end, expansion relative to the length of bone: (0) less than ½, (1) more than ½.

106. Coracoid, sternal end, shape: (0) symmetrical, (1) asymmetrical.

107. Coracoid, posterodorsal process: (0) absent, (1) present.

108. Clavicle: (0) present, (1) absent.

109. Clavicle, orientation: (0) anteromedial, (1) perpendicular, (2) posterior.

110. Clavicle, medial portion, shape: (0) strongly bowed anteriorly, (1) moderately bowed anteriorly, (2) straight or nearly so, (3) bowed posteriorly.

111. Clavicle, relationship with scapula: (0) overlaps anterior margin, (1) abuts pars acromialis, (2) fused.

112. Scapula, maximum width of glenoid fossa relative to maximum width of shaft: (0) more than 1, (1) between 1 and ½, (2) less than ½.

113. Scapula, medial cleft: (0) absent, (1) present.

114. Scapula, anterior margin, processes or crests: (0) absent, (1) ridge or a small knob on pars acromialis, (2) moderately crest on proximal half of shaft, (3) long lamina, (4) with a short crest strongly deflected anteroventrally to form a deep basin.

115. Cleithrum, overall shape in dorsal view: (0) lip of bone along the anterior margin of suprascapula, (1) lateral (scapular) margin straight, distinctly forked with anterior and posterior branches, (2) lateral margin may have a small notch at the anterior corner, distinct anterior branch, but posterolateral region of cleithrum plate-like, a small posterior branch may be present, (3) lateral margin with/out an ample notch, posterior region narrow, lacking a posterior branch, (4) extensive plate-like element investing almost entire dorsal surface of supraescapular cartilage.

**Forelimb**

116. Humerus, ventral condyle, diameter relative to total distal width at epicondyle level: (0) small (<0.58), (1) large (> 0.58).

117. Intercalary elements: (0) absent, (1) present.

118. Preaxial carpals (element Y and distal 2), configuration: (0) separate, (1) fused.

119. Postaxial carpals (ulnare and distals 3, 4 and 5), configuration: (0) all free, (1) ulnare and 3 free, 4+5, (2) ulnare free, 3+4+5, (3) ulnare+5, 3 and 4 free.

120. Prepollex, number and shape of prepollical elements: (0) one, spherical or elongate, (1) two, distal may be elongate, (2) three or more, spherical or elongate, (3) two, distal hypermorphic, (4) two, spine-like.

121. Fingers 1 and 2 (digits II and III), relative lengths: (0) II< III (1) II= III, (2) II> III.

122. Carpal torsion: (0) absent, (1) present.

**Pelvic Girdle**

123. Ilium, dorsal crest, development: (0) absent or low ridge, (1) moderately high (0.5 to 0.9x height of shaft), (2) high, well developed (as high as shaft or more).

124. Ilium, ventral acetabular expansion in acetabular view relative to height of acetabulum: (0) reduced (maximum length < 0.4), (1) moderately developed (maximum length > 0.4 and < 0.8), (2) well developed (maximum length >0.8).

125. Ilium, angle between iliac shaft and ventral acetabular expansion: (0) acute, (1) straight, (2) obtuse.

126. Ilium, dorsal acetabular expansion in acetabular view in relation to height of acetabulum: (0) reduced (maximum length <0.4), (1) moderately developed (maximum length >0.4 and < 0.8), (2) well developed (maximum length >0.8).

127. Ilium, ischiatic process: (0) absent, (1) present.

128. Ilium, dorsal prominence: (0) absent, (1) present.

129. Ilium, dorsal tubercle morphology: (0) low prominence, protuberance inconspicuous, (1) prominence scarcely discernible from iliac outline, elongate protuberance slightly projected laterally, (2) spike-like prominence, protuberance inconspicuous or not, (3) low prominence, rounded protuberance slightly laterally projected, (4) prominence scarcely discernible from iliac outline, elongate protuberance laterally projected.

130. Ilium, spiral groove: (0) absent, (1) present.

131. Ischium, posterodorsal expansion: (0) not developed, (1) well developed.

132. Ischium, posterior margin, shape: (0) convex, (1) straight, (2) concave.

133. Pubic region, mineralization: (0) cartilaginous, (1) mineralized.

134. Epipubis: (0) absent, (1) present.

**Hindlimb**

135. Femur, femoral crest: (0) absent, (1) present.

136. Femur, length relative to tibiofibula length: (0) shorter, (1) nearly equal, (2) longer.

137. Proximal tarsals (tibiale and fibulare), configuration: (0) separate, (1) proximally and distally fused, (2) completely fused.

138. Distal tarsals 3 and 2, configuration: (0) free, (1) fused.

139. Distal tarsal 1, as a discrete element: (0) absent, (1) present.

140. Prehallux, number and shape of prehallical elements: (0) one, spherical or elongate (1) two, spherical or elongate, (2) three or more, spherical or elongate, (3) two, distal hypermorphic axe-head-shaped, (4) three or more, axe-head-shaped.

141. Toe IV, terminal phalanx, general shape: (0) straight, (1) curved.

142. Toe IV, terminal phalanx, shape of distal end: (0) simple, (1) notched, (2) T-shaped, (3) Y-shaped, (4) clearly knobbed, (5) arrow-shaped.

**External Morphology**

143. Pupil shape: (0) vertical, (1) triangular, (2) horizontal, or mainly so.

Appendix S2. Taxa list

† marks extinct taxa, taxa names in bold represent the taxa added by this study. Nomina in () represent the one used in the dataset of Báez and Gómez (2018) that were synonymized with the correct nomen (following the taxonomy proposed by Dubois et al., 2021).

*Adelotus brevis*

*Allophryne ruthveni*

*Alytes obstetricans*

*Arariphrynus placidoi †*

*Arthroleptis adolfifriederici*

*Arthroleptella lightfooti*

*Ascaphus truei*

*Asterophrys riparius* (*Cophixalus riparius*)

*Atelognathus patagonicus*

*Aubria* *subsigillata*

*Baurubatrachus pricei †*

*Beelzebufo ampinga †*

*Bombina variegata*

*Bufo bufo*

*Cacosternum boettgeri*

*Cacosternum namaquense*

*Calyptocephalella gayi*

*Cornufer guentheri* (*Ceratobatrachus guentheri*)

*Ceratophrys calcarata*

*Ceratophrys cornuta*

*Ceratophrys cranwelli*

*Chacophrys pierottii*

*Conraua crassipes*

*Cratia gracilis †*

***Cretadhefdaa taouzensis †***

*Cycloramphus dubius*

*Dermatonotus muelleri*

*Eleutherodactylus inoptatus*

*Eurycephalella alcinae †*

*Flectonotus fitzgeraldi*

*Gastrotheca monticola*

*Heleioporus australiacus*

*Heleophryne natalensis*

*Hemiphractus fasciatus*

***Hungarobatrachus szukacsi*** *†*

*Hylambates verrucosus* (*Phlyctimantis verrucosus*)

*Hypsiboas pulchellus*

*Indirana phrynoderma*

*Lepidobatrachus asper*

*Lepidobatrachus laevis*

*Leptodactylus latrans*

*Limnonectes biythii*

*Litoria lesueurii*

*Megophrys montana*

*Melanophryniscus stelzneri*

*Mixophyes schevilli*

*Natalobatrachus bonebergi*

*Nyctibatrachus major*

*Odontobatrachus natator*

*Odontophrynus americanus*

*Oreobates discoidalis*

*Osteopilus dominicensis*

*Pelobates cultripes*

*Pelodytes punctatus*

*Peltophryne empusa*

*Petropedetes palmipes*

*Petropedetes vulpiae*

*Phrynobatrachus kreffti*

*Phrynomantis bifasciatus*

*Phyllomedusa sauvagii*

*Platyplectrum ornatum*

*Pleurodema cinereum*

*Pristimantis huicundo*

*Pristimantis wnigrum*

*Proceratophrys boiei*

*Ptychadena mascareniensis*

*Pyxicephalus adspersus*

*Rana temporaria*

*Ranoidea australis* (*Litoria Cyclorana australis*)

*Ranoidea longipes* (*Litoria Cyclorana longipes*)

*Rhinella arenarum*

*Scaphiopus couchii*

*Spea hammondi*

*Spea multiplicata*

*Strabomantis bufoniformis*

*Strabomantis ingeri*

*Strongolypus grayii*

*Telmatobius marmoratus*

*Telmatobius oxycephalus*

*Telmatobius verrucosus*

*Telmatobius hauthali*

*Telmatobius scrocchii*

*Telmatobufo venustus*

*Thaumastosaurus servatus †*

*Thoropa miliaris*

*Tomopterna tuberculosa*

*Uberabatrachus carvalhoi* *†*

Appendix S3. Nexus file containing the matrix used for all analyses.

Appendix S4. List of synapomorphies for selected clades for all analyses.

**Analysis under EW, ordered**

Cerotophryoidea+ *Uberabatrachus* + *Taxon A*: 61: 0–>1; 76: 1–>2; 125: 1–>2.

Ceraotphryoidea+ Taxon A: 6: 0–>1; 8: 0–>2; 12: 0–>1; 25: 1–>0; 49: 0–>2.

**Analysis under EW, excluding *Arariphrynus placidoi*, ordered**

‘Neobatrachia’ (excluding *Heleophryne*): 9: 0–>1; 114: 3–>0; 119: 0–>2; 130: 0–>1.

Hyperossified clade : 6: 0–>1; 10: 1–>2; 25: 1–>0; 61: 0–>2; 68: 1–>2; 121: 1–>2.

*Hungarobatrachus* + *Eurycephalella*+ *Calyptocephalella*: 8: 0–>2; 12: 0–>1; 119: 2–>1.

Taxon A + Ceratophryidae: 8: 0–>1; 29: 1–>0; 37: 0–>1; 61: 1–>2.
